# Through‐space interaction enables simultaneous enhancements of *k*_*r*_ and *k*_RISC_ in highly efficient spiro‐acridine based thermally activated delayed fluorescence emitter with acridone acceptor

**DOI:** 10.1002/smo.20240055

**Published:** 2024-12-01

**Authors:** Yongqiang Mei, Di Liu, Jiuyan Li, Min Xu, Jiahui Wang, Jing Jin, Lijuan Xie, Huihui Wan

**Affiliations:** ^1^ Frontier Science Center for Smart Materials School of Chemistry Dalian University of Technology Dalian China; ^2^ Frontier Science Center for Smart Materials College of Chemical Engineering Dalian University of Technology Dalian China; ^3^ Shandong Laboratory of Advanced Materials and Green Manufacturing at Yantai Yantai Economic and Technological Development Zone Yantai China; ^4^ Instrumental Analysis Center Dalian University of Technology Dalian China

**Keywords:** acridine donor, intramolecular through‐space interaction, low efficiency roll‐off, radiative rate constant, reverse intersystem crossing (RISC), thermally activated delayed fluorescence (TADF)

## Abstract

Most of acridine based thermally activated delayed fluorescence (TADF) emitters are characterized by advantageous reverse intersystem crossing (RISC) rate (*k*_RISC_s) due to the perpendicular orientation of the acridine donor to the acceptor moiety, but suffer from a poor radiation rate (*k*_*r*_) typically in the order of 10^6^ s^−1^. Herein, two sky blue TADF emitters 3,6‐DMAC‐AD‐Py and 3,6‐SFAC‐AD‐Py were developed by linking acridine (DMAC) and spiro‐fluorene‐acridine (SFAC) donors to 10‐(pyridin‐2‐yl)acridin‐9(10*H*)‐one (AD‐Py) acceptor. Larger SFAC and electron‐deficient pyridyl groups are deliberately incorporated in 3,6‐SFAC‐AD‐Py since the unique through‐space interaction between them is designed to drive the rotation of inner acridine ring in SFAC for enhancing frontier molecular orbitals overlap while keeping a decent TADF behavior. Thus, the *k*_*r*_ of 3,6‐SFAC‐AD‐Py is increased to 1.5 × 10^7^ s^−1^. Simultaneously, SFAC donors improve spin orbital coupling strength and reduce the energy gaps, generating *k*_RISC_ of 1.8 × 10^6^ s^−1^. This is the first acridine donor based TADF emitter realizing *k*_*r*_ of 10^7^ s^−1^ and *k*_RISC_ of 10^6^ s^−1^ by a through‐space interaction strategy. 3,6‐SFAC‐AD‐Py enables a highly efficient sky‐blue organic light‐emitting diode with a maximum external quantum efficiency (EQE) of 34.7% and Commission International de I'Eclairage coordinates of (0.19, 0.37). More importantly, the EQE still remained 27.6% and 16.9% at high brightness of 1000 and 10,000 cd m^−2^.

## Graphical abstract

Intramolecular through‐space interaction is designed for the first time in a thermally activated delayed fluorescence emitter to pull the appropriate rotation of the acridine donor to deviate from the perpendicular orientation, which solves the common problem of insufficient FMO overlap to simultaneously enhance the radiation transition and reverse intersystem crossing process and thus lead to a high EQE of 34.7% with quite slow efficiency roll‐off in blue organic light‐emitting diodes.

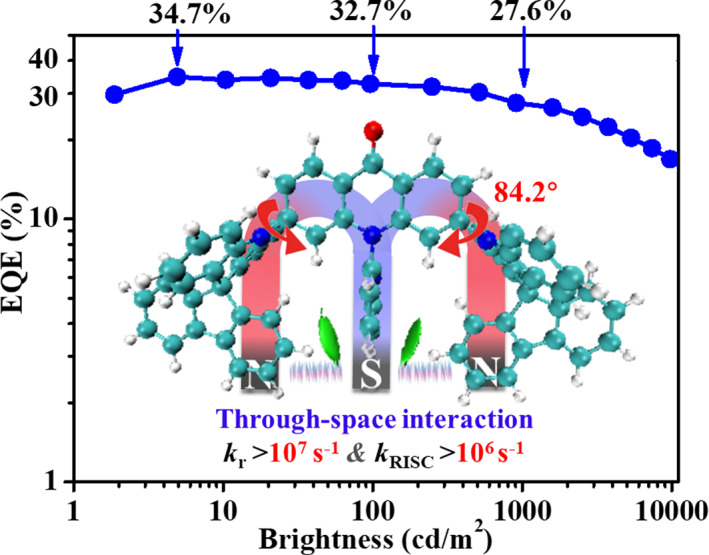

## 1 INTRODUCTION

Organic light‐emitting diodes (OLEDs) based on thermally activated delayed fluorescence (TADF) emitters have attracted increasing attention from both academic and industrial committees due to their advantageous merits of high efficiencies and low cost.[^1^, ^2^, ^3^, ^4^, ^5^, ^6^] Compared to the traditional fluorescent materials that can only utilize singlet excitons for radiation, TADF emitters can harvest non‐emissive triplet and emissive singlet excitons via efficient reverse intersystem crossing (RISC) process, achieving an internal quantum efficiency of nearly 100% in OLEDs.[^1^, ^2^, ^3^, ^4^, ^5^, ^6^, ^7^, ^8^, ^9^, ^10^, ^11^, ^12^, ^13^, ^14^, ^15^] According to the Fermi's golden rule that *k*_RISC_ is proportional to $\langle S\left|{\hat{H}}_{\text{SOC}}\right|T\rangle /∆E_{\text{ST}}$ ^[4]^ a fast and efficient RISC process relies on both tiny singlet‐triplet energy splitting (Δ*E*_ST_) and favorable spin‐orbital coupling (SOC) matrix element between triplet and singlet excited states. In addition to the efficient utilization of triplet excitons through RISC, a sufficiently high radiation rate constant *k*_*r*_ that directly denotes to a high photoluminescence (PL) quantum yield is essential to determine the overall performance of the TADF emitters in OLEDs.[^9^, ^15^, ^16^, ^17^, ^18^, ^19^, ^20^] Furthermore, the balance and fine trade‐off between the *k*_RISC_ and *k*_*r*_ are necessary to maintain a high external quantum efficiency (EQE) and slow efficiency roll‐off.

Theoretically, the Δ*E*_ST_ and SOC values of TADF emitters can be regulated by selecting suitable donors (D) and acceptors (A). Among various donor moieties, acridine and derivatives have been widely adopted in blue TADF emitters due to their large steric effect and moderate electron donating ability.[^1^, ^3^, ^4^, ^6^, ^7^, ^8^, ^9^, ^10^, ^11^, ^14^, ^17^, ^20^, ^21^, ^22^, ^23^] In 2019, Lee and Kwon et. al. reported a blue TADF emitter TDBA‐DI.^[21]^ Benefiting from the large steric hindrance between the DMAC donor and oxygen‐bridged boron acceptor, TDBA‐DI achieved a small Δ*E*_ST_ and fast *k*_RISC_ in the doped film and an EQE_max_ of 32.2% with Commission International de I'Eclairage (CIE; 0.14, 0.15) in the OLED. In 2021, Su et al. developed a sky blue TADF emitter DspiroS‐TRZ with 10*H*‐spiro[acridine‐9,9′‐thioxanthene] acceptor.^[22]^ The incorporation of thioxanthene into the DMAC donor adjusted the energy of the locally excited triplet state (^3^LE) to be close to the charge‐transfer singlet and triplet states (^1^CT and ^3^CT), facilitating the SOC and RISC processes. It exhibited EQE_max_ of 38.4% with CIE (0.18, 0.37) in OLED. However, many acridine based TADF emitters suffer from relatively low *k*_*r*_ values mainly caused by the nearly negligible overlap of HOMO and LUMO due to the almost perpendicular orientation of acridine ring to the acceptor. For example, the *k*_*r*_ in the order of 10^6^ s^−1^ or even lower have been detected for many acridine based TADF emitters.[^2^, ^3^, ^7^, ^12^] In order to facilitate the radiation to reach a reasonable balance with the RISC process, several typical design strategies were invented for acridine based materials in recent years. Tri‐spiral acridine skeletons were designed for constructing blue TADF emitters,[^5^, ^22^] which generally led to blue shifted emission relative to the parent acridine due to reduced electron donating ability and decreased *k*_RISC_ (typically below 10^6^ s^−1^). Increasing the conjugated structure is another modification method of acridine donor to enlarge the HOMO distribution of the whole molecule and thus improve the *k*_*r*_ values.[^8^, ^9^, ^10^] Electron donating groups were also ever attached onto the acridine to enhance the donor strength and finally enhance ICT extent and thus increase the *k*_*r*_ value.^[6]^ All the above optimization methods of *k*_*r*_ involve chemical structure modification, which usually involves tedious synthesis of acridine derivatives. Table S1 in Supporting Information S1 summarizes the typical TADF emitters containing acridine or derivated acridine donors reported in recent years and their key parameters. So far, there has been no report using physical strategies, such as intramolecular through‐space interaction, to enhance the *k*_*r*_ of acridine based TADF emitters so as to realize simultaneous high efficiency and low efficiency decay.

Likewise, the choice of an appropriate acceptor is equally important to achieve high efficiency for blue TADF emitters.[^24^, ^25^, ^26^, ^27^, ^28^, ^29^, ^30^, ^31^, ^32^, ^33^, ^34^, ^35^, ^36^] In 2023, we reported a series of blue TADF emitters based on 10‐phenylacridin‐9(10*H*)‐one (AD‐Ph, where acridin‐9(10*H*)‐one is also termed as acridone) and 10‐(pyridin‐2‐yl)acridin‐9(10*H*)‐one (AD‐Py) acceptors.[^24^, ^32^] Compared to the AD‐Ph,[^23^, ^33^, ^34^] the incorporation of pyridyl at 10‐site of acridone ring in AD‐Py brings about the formation of intramolecular H‐bond between the pyridyl N atom and the 4‐ or 5‐site H atom of acridone ring, which not only enhances molecular rigidity to restrain nonradiative process but also raises the ^3^LE energy to be close to the ^1^CT and ^3^CT states and leads to a strong SOC and fast RISC process, finally making AD‐Py unit a more potential acceptor for blue TADF. As a result, almost all the AD‐Py based blue TADF emitters exhibited maximum external quantum efficiencies (EQEs_max_) exceeding 30%, updating the efficiency record for the TICT type blue TADF emitters so far. However, it was really a pity that remarkable efficiency roll‐off was still observed for those emitters.^[24]^

**FIGURE 1:**
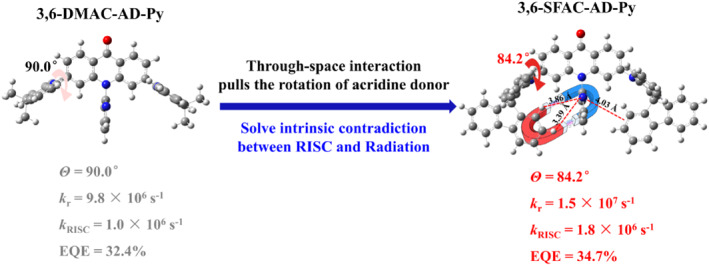
The molecular design strategy of 3,6‐DMAC‐AD‐Py and 3,6‐SFAC‐AD‐Py.

In this work, two new sky blue TADF emitters, namely 10‐(pyridin‐2‐yl)‐3,6‐di(9,9‐dimethyl‐9,10‐dihydroacridine‐10‐yl)acridin‐9(10*H*)‐one (3,6‐DMAC‐AD‐Py) and 10‐(pyridin‐2‐yl)‐3,6‐di(10*H*‐spiro[acridine‐9,9′‐fluoren]‐10‐yl)acridin‐9(10*H*)‐one (3,6‐SFAC‐AD‐Py) (Figure 1), were designed and synthesized by grafting two dimethylacridine (DMAC) and spiro‐fluorene‐acridine (SFAC) donors at 3,6‐sites of the AD‐Py acceptor, respectively. AD‐Py was selected with expectation that the large molecular rigidity caused by the intramolecular H‐bond between the pyridyl N atom and the 4‐ or 5‐site H atom of acridone ring strongly restricts the nonradiative transitions and the closely aligned ^3^LE ≈^1^CT ≈^3^CT states and the strong SOC favor fast RISC process. In particular, SFAC was incorporated as donors in 3,6‐SFAC‐AD‐Py with expectation that the SFAC group that is longer than the parent DMAC group can form effective through‐space interaction with the pyridyl unit of the AD‐Py acceptor based on their individual electron donating and deficient natures, which will force the acridine plane to rotate to some extent from its original orthogonal orientation. In such a way, sufficient HOMO‐LUMO separation (on inner acridine ring of SFAC donors and acridone acceptor) and appropriate HOMO‐LUMO overlap were simultaneously realized in 3,6‐SFAC‐AD‐Py, which thus increases the *k*_*r*_ to 1.5 × 10^7^ s^−1^ while keeping *k*_RISC_ as high as 1.8 × 10^6^ s^−1^, in contrast to 9.8 × 10^6^ and 1.0 × 10^6^ s^−1^ for 3,6‐DMAC‐AD‐Py. This is the first example of acridine based TADF emitter with simultaneously high *k*_*r*_ in the order of 10^7^ s^−1^ and *k*_RISC_ of 10^6^ s^−1^ achieved by means of intramolecular through‐space interaction between functional building blocks. As a result of balanced *k*_*r*_ and *k*_RISC_, the sky blue OLED of 3,6‐SFAC‐AD‐Py realized an EQE_max_ of 34.7% with CIE coordinates of (0.19, 0.37), which is higher than that of 3,6‐DMAC‐AD‐Py (EQE_max_ of 32.4%) with CIE (0.22, 0.43), and also among the best values of blue TICT‐TADF emitters. Furthermore, the 3,6‐SFAC‐AD‐Py device showed very low efficiency roll‐offs of 6% and 20% at brightness of 100/1000 cd m^−2^, respectively. Remarkably, the EQE remained 16.9% at 10,000 cd m^−2^.

## 2 RESULTS AND DISCUSSION

### 2.1 Synthesis and electrochemical properties

**SCHEME 1:**
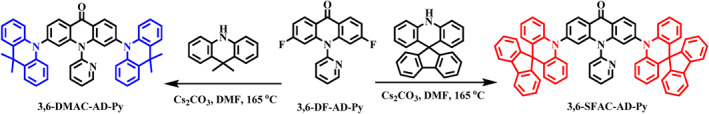
Chemical structures and synthetic routes of 3,6‐DMAC‐AD‐Py and 3,6‐SFAC‐AD‐Py.

3,6‐DMAC‐AD‐Py and 3,6‐SFAC‐AD‐Py were synthesized through a nucleophilic substitution reaction between the DMAC/SFAC and fluorinated intermediate 3,6‐DF‐AD‐Py in the presence of cesium carbonate in anhydrous N,N‐dimethyl‐formamide at 165°C (Scheme 1).[^23^, ^24^, ^33^, ^34^, ^37^] The synthetic and structure characterization details are provided in Supporting Information S1. The HOMO and LUMO levels were determined by cyclic voltammetry measurements (Figure S1 in Supporting Information S1) as −5.29/−2.84 and −5.46/−2.92 eV (Table 1) for 3,6‐DMAC‐AD‐Py and 3,6‐SFAC‐AD‐Py, respectively. The deeper HOMO level of 3,6‐SFAC‐AD‐Py should originate from the less electron donating ability of the SFAC group, which will definitely favor the blue color purity.

**TABLE 1:** Experimentally obtained parameters for 3,6‐DMAC‐AD‐Py and 3,6‐SFAC‐AD‐Py.

Compounds	*λ*_abs_ ^a^ (nm)	*λ*_em_ ^a^ (nm)	*E*_S_ ^b^ (eV)	*E*_T_ ^b^ (eV)	Δ*E*_ST_ ^b^ (eV)	HOMO^c^ (eV)	LUMO^c^ (eV)	*E*_*g*_ ^c^ (eV)
3,6‐DMAC‐AD‐Py	372/388	511	2.46	2.46	0	−5.29	−2.84	2.45
3,6‐SFAC‐AD‐Py	371/390	490	2.54	2.54	0	−5.46	−2.92	2.54

^a^ Absorption and fluorescence peak wavelengths in dilute toluene solutions at room temperature.

^b^ Energies and energy difference estimated from the LT‐PL and LT‐PH spectra at 77 K in doped PPF films (7 wt%).

^c^ Determined from electrochemical measurements.

### 2.2 Theoretical calculations

**FIGURE 2:**
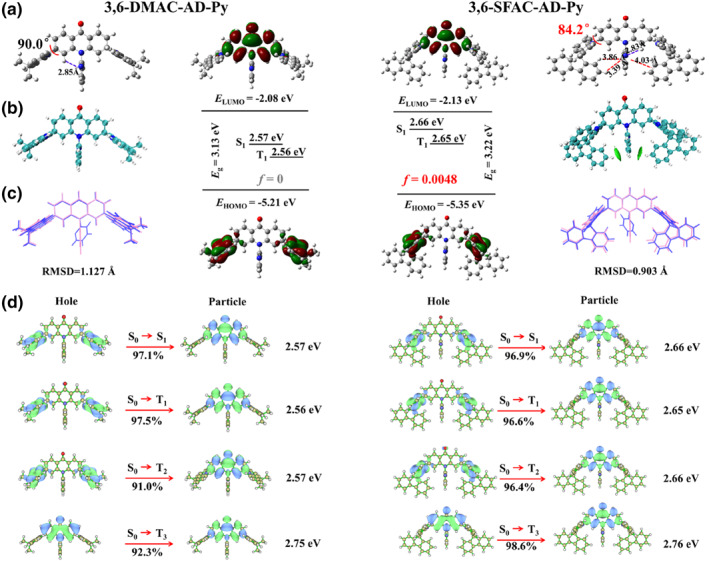
FMOs distributions and levels and optimized S_0_ geometries (a), visualization of through‐space interaction calculated based on optimized S_0_ geometries (b), root mean square displacement between optimized S_0_ (mauve) and S_1_ (blue) geometries (c), and calculated natural transition orbitals (d) for 3,6‐DMAC‐AD‐Py and 3,6‐SFAC‐AD‐Py. FMOs, frontier molecular orbitals.

The frontier molecular orbital (FMO) distributions and excited states energy levels of 3,6‐DMAC‐AD‐Py and 3,6‐SFAC‐AD‐Py were investigated by density functional theory (DFT) and time‐dependent DFT (TD‐DFT) calculations using the Gaussian 16 program with the B3LYP‐D3(BJ)/6‐311g (d,p) method and are shown Figure 2. In these two AD‐Py based molecules, the intramolecular H‐bond was verified by the distance between the pyridyl N atom and the H atom at ‐ or 5‐site of the acridine ring as short as 2.83 or 2.85 Å, which are consistent with the detected distances in single crystals of other TADF emitters with AD‐Py as acceptor.^[24]^ As shown in Figure 2a and Figure S2 in Supporting Information S1, for both 3,6‐DMAC‐AD‐Py and 3,6‐SFAC‐AD‐Py, the HOMO is mainly located on the inner acridine ring of the corresponding DMAC or SFAC donors, while the LUMO distributes exclusively on the central acridone (AD) ring. Interestingly, the HOMO‐2 and LUMO+1 of 3,6‐SFAC‐AD‐Py are localized on fluorene and pyridyl groups (Figure S3 in Supporting Information S1), respectively, suggesting fluorene and pyridyl groups have electron‐donating and ‐withdrawing ability to some extent and the mutual attraction effect betweem them is possible. In order to verify such interaction in 3,6‐SFAC‐AD‐Py, a pyridyl‐free reference compound 3,6‐SFAC‐AD (10‐phenyl‐3,6‐di(10*H*‐spiro[acridine‐9,9′‐fluoren]‐10‐yl)acridin‐9(10*H*)‐one) with phenyl substituted AD (AD‐Ph) as acceptor (structure shown in Figure S3 in Supporting Information S1) was calculated under identical conditions for comparison. As expected, the optimized geometries of 3,6‐DMAC‐AD‐Py (Figure 2a) and the reference compound 3,6‐SFAC‐AD (Figure S3 in Supporting Information S1) exhibit the large torsion angles of nearly 90° between the AD plane and the adjacent acridine ring of the DMAC or SFAC donors, as in many acridine based analogues.[^2^, ^5^, ^7^, ^9^] In contrast, this dihedral angle in 3,6‐SFAC‐AD‐Py molecule decreases to 84.2° with a relatively large *f* (0.0048), indicating that the inner acridine ring of the SFAC donors has deviated from the orthogonal orientation relative to the AD acceptor. In addition, in 3,6‐SFAC‐AD‐Py molecule (Figure 2a and Figure S3 in Supporting Information S1), the peripheral fluorene rings show the edge‐to‐face packing style to the pyridyl ring, with the distances of 4.03 Å from the spiro‐fluorene edge (C‐C bond) to the pyridyl face and of 3.39–3.86 Å from the H atoms of fluorene ring to pyridyl face, both which are shorter than the corresponding values (4.42 and 3.57–3.84 Å) in the pyridyl‐free reference compound 3,6‐SFAC‐AD. All these distances are within the effective van der Waals distance, indicating the possible through‐space interaction that may belong to a type of weak attraction force between the peripheral fluorene moiety and the pyridyl ring in 3,6‐SFAC‐AD‐Py molecule.[^25^, ^29^, ^36^] In order to explore the existence of such intramolecular interaction particularly in 3,6‐SFAC‐AD‐Py, the independent gradient model (IGM) in the light of the optimized ground state geometry was studied in Multiwfn for both emitters, and the results are shown in Figure 2b. Such kind of strong through‐space interaction within this edge‐to‐face packing configuration is exclusively revealed between the fluorene moiety to the pyridyl plane in 3,6‐SFAC‐AD‐Py, as clearly visualized by the presence of the green cloud in Figure 2b. Evidently, the through‐space interaction successfully drove the rotation of the acridine plane by certain angle to reach appropriate HOMO‐LUMO overlap was realized in 3,6‐SFAC‐AD‐Py, which should be favorable for more efficient radiative transition. Simultaneously, 3,6‐SFAC‐AD‐Py showed a lower root mean square displacement (RMSD) value of 0.903 Å than 3,6‐DMAC‐AD‐Py (1.127 Å), proving that the unique through‐space interaction between fluorene and pyridyl groups could further increase the rigidity of 3,6‐SFAC‐AD‐Py to suppress the nonradiative processes for the higher PLQY and lower *k*_nr_.

Additionally, the natural transition orbitals (NTOs) and SOC matrix elements were calculated^[38]^ and are shown in Figure 2d and Table S2 in Supporting Information S1. For both molecules, their S_1_ states are characterized by the charge transfer (^1^CT) feature, and the S_1_ state of 3,6‐SFAC‐AD‐Py has higher energy (2.66 eV) than that (2.57 eV) of 3,6‐DMAC‐AD‐Py, as predicted by its deeper HOMO level (Figure S1 in Supporting Information S1). This is reasonable because the SFAC donor has weaker electron donating ability than DMAC and thus causes a relatively weaker CT effect. The T_1_ and T_2_ states that are both ^3^CT states have degenerated energies, and also have almost identical energies to the corresponding S_1_ (^1^CT) state, leading to the almost zero ${\Delta E}_{S_{1}T_{1}}$. The calculated T_3_ states of 3,6‐DMAC‐AD‐Py and 3,6‐SFAC‐AD‐Py correspond to the ^3^LE state of the AD‐Py acceptor. Based on the higher S_1_ (^1^CT) energy and identical T_3_ levels, 3,6‐SFAC‐AD‐Py reveals a smaller ${\Delta E}_{S_{1}T_{3}}$ gap (0.10 eV) than 3,6‐DMAC‐AD‐Py (0.18 eV). Furthermore, due to the appropriate contribution of the SFAC group to the features of excited states, 3,6‐SFAC‐AD‐Py exhibited a larger SOC of 1.111 cm^−1^ between ^3^LE (T_3_) and ^1^CT (S_1_) than that of 3,6‐DMAC‐AD‐Py (0.933 cm^−1^, Table S2 in Supporting Information S1). Both reduced ${\Delta E}_{S_{1}T_{3}}$ and enhanced SOC values are anticipated to lead to a faster RISC process in the 3,6‐SFAC‐AD‐Py.

### 2.3 Photophysical properties

**FIGURE 3:**
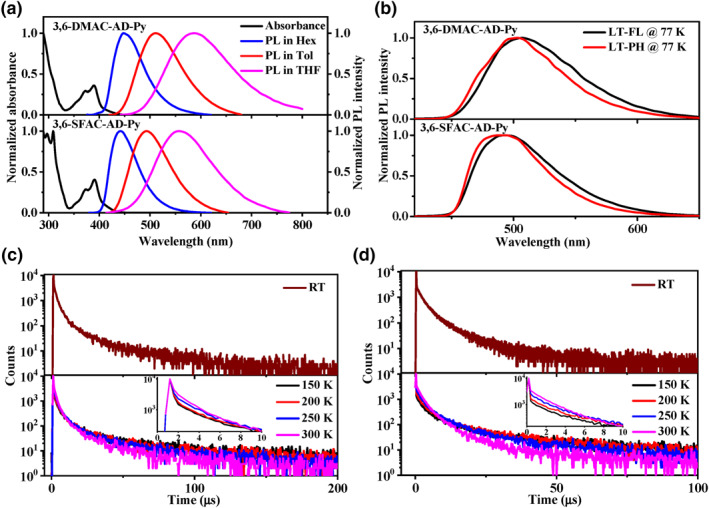
Absorption and PL spectra in dilute solutions at room temperature (a), LT‐PL and LT‐PH spectra in doped 2,8‐bis(diphenylphosphoryl)dibenzo[b, d]furan films (7 wt%) at 77 K (b), time‐resolved transient and the temperature‐dependent PL decay curves in doped films (c, d, enlarged figure in first 10 µs are inserted) for 3,6‐DMAC‐AD‐Py and 3,6‐SFAC‐AD‐Py. LT, low‐temperature; PH, phosphorescence; PL, photoluminescence.

The ultraviolet‐visible (UV‐vis) absorption in dilute toluene solutions (10^−5^ M) and PL spectra in different solvents at room temperature for both emitters are presented in Figure 3a and the relevant data are summarized in Table 1. The high‐energy absorption peaks at 290–320 nm should be ascribed to the *n*–π* and *π*–π* transitions of the DMAC and SFAC donors,[^1^, ^3^] while the broad trailing absorption band with relatively weak intensity at around 350–450 nm should originate from the *n*–π* and *π*–π* transitions of AD‐Py units with certain contribution from the CT transition between D and A units.[^23^, ^24^, ^33^, ^34^] 3,6‐DMAC‐AD‐Py and 3,6‐SFAC‐AD‐Py show broad and structureless sky blue fluorescence peaking at 511 and 490 nm with full width at half maximum (FWHM) of 103 nm (0.46 eV) and 90 nm (0.42 eV), respectively. On one hand, the narrower FWHM of 3,6‐SFAC‐AD‐Py should be due to the through‐space interaction (Figure 2b) that effectively restrains molecular geometrical changes between S_0_ and S_1_ states and vibrational relaxation in the S_1_ state. On the other hand, the deeper blue spectrum of 3,6‐SFAC‐AD‐Py should be due to the weaker electron‐donating property of the SFAC group than DMAC.^[1]^ Moreover, with increasing solvent polarity, 3,6‐DMAC‐AD‐Py and 3,6‐SFAC‐AD‐Py exhibit remarkable red shift with broadening of the spectra, confirming their typical ICT characters.[^5^, ^6^, ^7^, ^8^, ^9^, ^10^]

The low‐temperature fluorescence (LT‐FL) and phosphorescence (PH) spectra of these emitters were measured for their doping films with 2,8‐bis(diphenylphosphoryl)dibenzo[b, d]furan (PPF) host (7 wt%) at 77 K, and are shown in Figure 3b. Their LT‐FL and LT‐PH spectra exhibit featureless broad profiles, demonstrating that their S_1_ and T_1_ states are mainly CT transition characterized, consistent with the theoretical simulation results (Figure 2d). From the onset wavelength of the LT‐FL and LT‐PH spectra, the S_1_ and T_1_ energies were determined and summarized in Table 1. Similar to theoretical prediction (Figure 2d), 3,6‐SFAC‐AD‐Py exhibits higher S_1_ and T_1_ energies than 3,6‐DMAC‐AD‐Py, and both emitters exhibit zero ${\Delta E}_{S_{1}T_{1}}$. The fluorescence and phosphorescence of the fragment compound AD‐Py (acceptor) were also measured (Figure S4 in Supporting Information S1) and its T_1_ level was determined to be 2.64 eV, which corresponds to the ^3^LE (T_3_) state of these emitters (Figure 2d).

**FIGURE 4:**
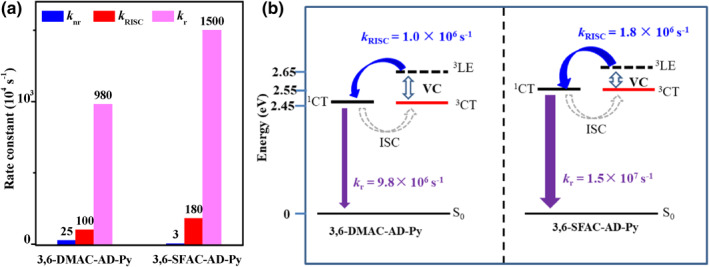
Comparison of the transition rate constants *k*_nr_s, *k*_r_s, and *k*_RISC_s (a), and schematic illustration of decay, up‐conversion, and intersystem crossing processes (b) of 3,6‐DMAC‐AD‐Py and 3,6‐SFAC‐AD‐Py.

To confirm their TADF characters, the PL transient decay behaviors of the doped film of 3,6‐DMAC‐AD‐Py and 3,6‐SFAC‐AD‐Py in PPF (7 wt%) were measured and illustrated in Figure 3c,d and Figures S3, S4 in Supporting Information S1 and the data are summarized in Table 2. The double‐exponential decay curves are displayed including prompt and delayed components with lifetimes (*τ*_PF_ and *τ*_DF_) of 21.5 ns/4.6 µs for 3,6‐DMAC‐AD‐Py and 14.9 ns/3.3 µs for 3,6‐SFAC‐AD‐Py, respectively. The identical spectral profiles of the delayed component to the prompt fluorescence (Figure S6 in Supporting Information S1) and the positive temperature dependence of the PL intensity in the short time range (e.g. the first 10 µs) with increasing temperature (Figure 3c,d) combine to confirm the TADF nature of the delayed component for both emitters. The PLQYs of the two emitters in films were determined as 97.5% for 3,6‐DMAC‐AD‐Py and 99.8% for 3,6‐SFAC‐AD‐Py, respectively. With all the above data, the radiative transition rate constant *k*_r_s, the non‐radiative transition rate constant *k*_nr_s and the RISC rate constants *k*_RISC_s of the two emitters were calculated following the methods described in Supporting Information S1, and the data are summarized in Table 2 and compared in Figure 4a. On one hand, compared to 3,6‐DMAC‐AD‐Py (*k*_nr_ = 25.1 × 10^4^ s^−1^ and *k*_*r*_ = 9.8 × 10^6^ s^−1^), the slower *k*_nr_ (2.9 × 10^4^ s^−1^) and faster *k*_*r*_ (1.5 × 10^7^ s^−1^) of 3,6‐SFAC‐AD‐Py should benefit from its highly rigid configuration caused by the unique through‐space interaction between the peripheral fluorene and pyridine rings and high *f* value as aforementioned. On the other hand, a shorter *τ*_DF_ (3.3 µs) and faster *k*_RISC_ (1.8 × 10^6^ s^−1^) of 3,6‐SFAC‐AD‐Py owing to the small energy gap and larger SOC values among excited states (Table S2 in Supporting Information S1) indicate highly efficient utilization of the triplet excitons for light emission. Both favorable *k*_*r*_ and *k*_RISC_ (Figure 4b) predict the advantageous performance of 3,6‐SFAC‐AD‐Py in OLEDs.

**TABLE 2:** Photophysical data of the investigated molecules in doped films (7 wt% in 2,8‐bis(diphenylphosphoryl)dibenzo[b, d]furan) at room temperature.^a^

Compounds	Φ_PL_ (%)	Φ_PF_ (%)	Φ_DF_ (%)	*τ*_PF_ (ns)	*τ*_DF_ (μs)	*k*_*r*_ (10^6^ s^−1^)	*k*_nr_ (10^4^ s^−1^)	*k*_ISC_ (10^6^ s^−1^)	*k*_RISC_ (10^6^ s^−1^)
3,6‐DMAC‐AD‐Py	97.5	21.1	76.4	21.5	4.6	9.8	25.1	36.4	1.0
3,6‐SFAC‐AD‐Py	99.8	21.5	78.3	14.9	3.3	15.0	2.9	52.5	1.8

^a^ *k*_*r*_, *k*_nr_, *k*_ISC_, and *k*_RISC_ represent the rate constant of radiative transition, non‐radiative transition, intersystem crossing, and reverse intersystem crossing, respectively; Φ_PL_, Φ_PF_, Φ_DF_, *τ*_PF_, and *τ*_DF_ represent the quantum yield of the total emission, the PF, the DF, and the average lifetime of the PF and DF, respectively.

### 2.4 Electroluminescence

**FIGURE 5:**
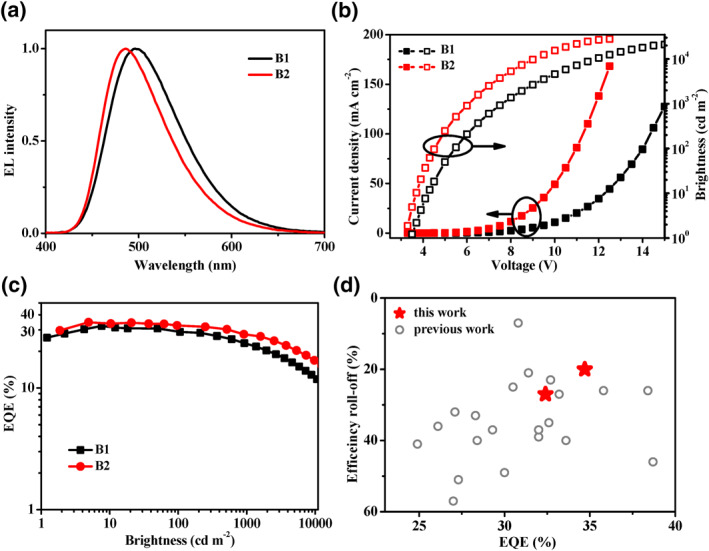
EL spectra (a), *J‐V‐B* characteristics (b) and efficiency curves (c) of thermally activated delayed fluorescence‐organic light‐emitting diodes B1 and B2, and comparison of EQE and efficiency roll‐off under 1000 cd m^−2^ between the present devices B1 and B2 and those reported blue emitters (480–500 nm, EQE >25%) in literatures (d). EQE, external quantum efficiency.

To better evaluate the electroluminescence (EL) performance of both blue TADF materials, multiple‐layer OLEDs were fabricated with structure of indium tin oxide/poly(3,4‐ethylenedioxythiophene:polystyrene sulfonate (PEDOT:PSS, 40 nm)/1,1‐bis[(di‐4‐tolylamino)phenyl]cyclohexane (TAPC, 20 nm)/4,4,4‐tris(N‐carbazolyl)triphenylamine (TCTA, 5 nm)/1,3‐bis(N‐carbazolyl)benzene (mCP, 5 nm)/PPF:TADF emitter (20 nm, 7 wt%)/PPF (5 nm)/1,3,5‐tris(3‐pyridyl‐3‐phenyl)benzene (TmPyPB, 40 nm)/LiF (1 nm)/Al (200 nm) (devices B1 and B2 for 3,6‐DMAC‐AD‐Py and 3,6‐SFAC‐AD‐Py, respectively). The device architecture, energy level diagram and chemical structures of the used materials in these devices are shown in Figure S7 in Supporting Information S1. PEDOT:PSS and TAPC were employed as the hole‐injecting and ‐transporting layers, respectively. LiF and TmPyPB served as the electron‐injecting and ‐transporting layers, respectively. TCTA, mCP, and PPF were adopted as exciton‐blocking layers to confine excitons within the emitting layer. Furthermore, the PPF was chosen as host to prevent reverse energy transfer from the dopant to the host, based on a higher T_1_ energy level reaching 3.0 eV.^[5a]^ The EL spectra, current density‐voltage‐brightness (*J‐V‐B*) characteristics and efficiency curves are illustrated in Figure 5 and Figure S7c in Supporting Information S1, and all the related EL data are summarized in Table 3.

**TABLE 3:** EL Performances of devices B1 and B2.

Devices	*V*_on_ (V)	CE (cd A^−1^)	PE (lm W^−1^)	EQE^a^ (%)	*λ*_EL_ (nm)	FWHM (nm)	CIE (*x*, *y*)
B1	3.5	83.5	64.0	32.4/28.9/23.4	496	90	0.22, 0.43
B2	3.3	78.8	70.7	34.7/32.7/27.6	486	79	0.19, 0.37

Abbreviations: CE, current efficiency; CIE (*x*, *y*), Commission International de I'Eclairage coordinates; EL, electroluminescence; EQE, external quantum efficiency; FWHM, full width at half maximum; PE, power efficiency; *V*_on_, turn‐on voltage at a brightness of 1 cd m^−2^; *λ*_EL_, EL peak wavelength.

^a^ Order of measured values: maximum, then at 100 and 1000 cd m^−2^.

As shown Figure 5a, the devices B1 and B2 display single sky‐blue emission with the EL peaks of 496 and 486 nm, CIE coordinates of (0.22, 0.43) and (0.19, 0.37) and FWHM of 90 and 79 nm, respectively. The 3,6‐DMAC‐AD‐Py based device B1 achieved an EQE_max_ of 32.4% with a maximum current efficiency (CE_max_) of 83.5 cd A^−1^ and power efficiency (PE_max_) of 64.0 lm W^−1^. Noteworthily, the 3,6‐SFAC‐AD‐Py based device B2 exhibited a state‐of‐the‐art EL performance with an EQE_max_ of 34.7%, CE_max_ of 78.8 cd A^−1^ and PE_max_ of 70.7 lm W^−1^, which are among the highest efficiencies for TADF‐OLED with similar EL emission peaks from 480 to 500 nm (Figure 5d). More importantly, the EQE still maintained at 32.7% and 27.6% at brightness of 100 and 1000 cd m^−2^, which correspond to the efficiency roll‐offs of 6% and 20% from the maximum value (EQE_max_), respectively. Remarkably, the EQE remained at 16.9% even at an extremely high brightness of 10,000 cd m^−2^. As shown in Figure 5d, among the reported sky blue TADF‐OLEDs with similar EQEs range (>25%), the efficiency roll‐off of the present 3,6‐SFAC‐AD‐Py based device B2 is quite low.[^4^, ^5^, ^8^, ^9^, ^10^, ^11^, ^12^, ^13^, ^14^, ^15^, ^16^, ^17^, ^18^, ^19^, ^20^, ^21^, ^22^, ^25^, ^29^] Such excellent performance and low efficiency roll‐off should be attributed to the high PLQY, fast *k*_*r*_ and *k*_RISC_. Enhancing *k*_*r*_ (>10^7^ s^−1^) while maintaining a high *k*_RISC_ (>10^6^ s^−1^) can help to build a superior balance between *k*_*r*_ and *k*_RISC_, contributing to a fast conversion process between singlet and triplet excitons and suppressing singlet‐singlet annihilation (SSA) and triplet–triplet annihilation.[^2^, ^7^, ^9^] Therefore, the rapid radiative decay and RISC process are equally important for TADF emitters to achieve simultaneously high EQE and low efficiency roll‐off.

## 3 CONCLUSION

Many TADF molecules containing acridine donors suffer from inferior *k*_*r*_ (typically in the order of 10^6^ s^−1^) due to orthogonal orientation and complete HOMO/LUMO separation despite excellent *k*_RISC_. Herein, two novel sky blue TADF emitters 3,6‐DMAC‐AD‐Py and 3,6‐SFAC‐AD‐Py were developed with 10‐pyridyl decorated acridone (AD‐Py) as acceptor and acridine (DMAC) or spiro‐acridine (SFAC) as donors. Particularly in 3,6‐SFAC‐AD‐Py, the unique intramolecular through‐space interaction between the peripheral fluorene moiety of SFAC donors and the pyridyl ring of the AD‐Py acceptor, that is, mutual attraction due to their electron‐rich and ‐deficient features, successfully pulled the rotation of inner acridine ring and led to appropriate FMO overlapping in 3,6‐SFAC‐AD‐Py. As a direct result, the *k*_*r*_ was increased from 9.8 × 10^6^ to 1.5 × 10^7^ s^−1^ while keeping a sufficiently high *k*_RISC_ of 1.8 × 10^6^ s^−1^. This is the first report to optimize *k*_*r*_ of acridine based TADF emitters by means of physical strategy, that is, through‐space interaction between functional building blocks, in contrast to the tedious chemical structure modifications in literature reports. By virtue of balanced *k*_*r*_ and *k*_RISC_, 3,6‐SFAC‐AD‐Py realized a very high EQE_max_ of 34.7% in simple‐structure blue OLED with impressive low efficiency roll‐off. Even at high brightness of 1000 and 10,000 cd m^−2^, the EQE remained as high as 27.6% and 16.9%, which were still higher than the maximum efficiencies of many similar blue TADF‐OLEDs. This work demonstrates a new practical strategy to simultaneously optimize *k*_*r*_ and *k*_RISC_ of blue TADF materials and realize high efficiencies and low efficiency roll‐offs in OLEDs.

## CONFLICT OF INTEREST STATEMENT

The authors declare no conflicts of interest.

## ETHICS STATEMENT

No animal or human experiments were involved in this study.

## Supporting information

Supporting Information S1

## Data Availability

Supporting information is available from the Wiley Online Library or from the author.
