# Supplementary material for: Through‐space interaction enables simultaneous enhancements of k r and k RISC in highly efficient spiro‐acridine based thermally activated delayed fluorescence emitter with acridone acceptor
Source: Smart Mol. 2024 Dec 1;3(4):e20240055. doi: 10.1002/smo.20240055 (PMC12755221; doi:10.1002/smo.20240055)
Supplement: Supplementary file 1 — Supporting Information S1 [file SMO2-3-e20240055-s001.docx]

**Supporting information**

**For**

**Through-Space Interaction Enables Simultaneous Enhancements of *k*_r_ and *k*_RISC_ in Highly Efficient Spiro-Acridine based TADF Emitter with Acridone Acceptor**

Yongqiang Mei,^1,2^ Di Liu,^1^ Jiuyan Li,^2,3^ Min Xu,^2^ Jiahui Wang,^2^ Jing Jin,^1^ Lijuan Xie,^1^ Huihui Wan^4^

^1^ Frontier Science Center for Smart Materials, School of Chemistry, Dalian University of Technology, 2 Linggong Road, Dalian, 116024, China

^2^ Frontier Science Center for Smart Materials, College of Chemical Engineering, Dalian University of Technology, 2 Linggong Road, Dalian, 116024, China

^3^ Shandong Laboratory of Advanced Materials and Green Manufacturing at Yantai, Yantai Economic and Technological Development Zone, 300 Changjiang Road, Yantai, China

^4^ Instrumental Analysis Center, Dalian University of Technology, Dalian, 116024, China

**Correspondence**

Di Liu and Jiuyan Li

E-mail: [liudi@dlut.edu.cn](mailto:liudi@dlut.edu.cn) and [jiuyanli@dlut.edu.cn](mailto:jiuyanli@dlut.edu.cn)

**1. Experimental Section**

**General information:** The ^1^H NMR and ^13^C NMR were measured by a Bruker Avance Ⅲ 400 and 101 MHz and Bruker Avance Ⅲ 500 and 126 MHz spectrophotometer. The mass spectra were obtained from HP1100LC/MSD MS spectrometer. UV-vis absorption and fluorescence spectra measurements were performed on a Perkin-Elmer Lambda 650 and a Hitachi F-7000 fluorescence spectrometer, respectively. Photoluminescence quantum yields (PLQYs) of films were recorded on HAMAMATSU absolute PL quantum yield spectrometer C11347. The PL spectra in different solvents, transient fluorescence decays, low temperature fluorescence and phosphorescence spectra were measured by using Edinburgh Instruments FLS1000 spectrometer. Cyclic voltammetry was carried out on Electrochemical workstation (CHI610E). The prompt fluorescence lifetimes were measured by the time correlated single photon counting (TCSPC) as data acquisition technique using a 375 nm LED as the excitation source at room temperature. And the delayed fluorescence lifetimes were measured through a multi-channel scaling (MCS) as data acquisition technique using a 365 nm LED as the excitation source at room temperature. The Lifetimes were calculated from the following equation:

$$\tau=\frac{B_{1}\tau_{1}^{2}+B_{2}\tau_{2}^{2}+B_{3}\tau_{3}^{2}}{B_{1}\tau_{1}+B_{2}\tau_{2}+B_{3}\tau_{3}}$$

The ground state geometries were optimized by DFT method using B3LYP-D3(BJ) functional at the level 6-311G(d,p) basis set employing Gaussian 16 software package. The corresponding excited-state energies, oscillator strengths (*f*) and natural transition orbitals (NTOs) were calculated using TD-DFT method (B3LYP-D3(BJ)/6-311G(d,p)) according to the optimized ground state geometries. The SOC matrix elements were acquired by ORCA 4.2.1 software package using B3LYP-D3(BJ) functional at the level 6-311G(d,p) basis set.^[1]^ The related HOMO, LUMO and NTOs were analyzed and then plotted through Multiwfn 3.8 and VMD.^[2]^

**OLED fabrication and measurements:** Before evaporating organic film and OLEDs, the ITO glass substrates were pre-cleaned with acetone, isopropanol detergents and deionized water successively and treated by oxygen plasma for 30 min. The substrate was then transferred into a deposition chamber. Devices were prepared by evaporating organic layers at a rate of 1.0 Å s^−1^ onto the ITO substrate sequentially at a pressure below 1×10^−4^ Pa. The doping concentration was accurately controlled by adjusting the different evaporation rate of the host and the dopant. 1 nm LiF and 200 nm Al were deposited for use as the electron injection layer and cathode, respectively. The EL spectra and CIE coordinates of the devices were measured with a PR705 photometer. The current density (J)-voltage (V) and brightness (B)-voltage (V) curves of the devices were recorded using a Keithley 2400 SMU and a Konica Minolta Chroma Meter CS-200.

**Quantum calculations**^[3,4]^

*k*_p_=1/*τ*_PF_

*k*_d_=1/*τ*_DF_

*k*_r_=*Φ*_PF_/*τ*_PF_

*Φ*_PL_=*k*_r_/(*k*_r_+*k*_nr_)

*Φ*_PF_=*k*_r_/(*k*_r_+*k*_ISC_ +*k*_nr_)

*k*_RISC_ = *k*_p_*k*_d_*Φ*_DF_/*k*_ISC_*Φ*_PF_

*k*_p_, *k*_d_, *k*_r_, *k*_nr_, *k*_ISC_, *k*_RISC_ represent the rate constants of prompt process, delay process, radiation, non-radiation, intersystem crossing, and reverse intersystem crossing, respectively. *Φ*_PL_, *Φ*_PF_, *Φ*_DF_, *τ*_PF_, and *τ*_DF_ represent total PLQY, quantum yield of the prompt component, quantum yield of the delayed component, average lifetimes of the prompt and delayed components, respectively.

**Scheme 1** Chemical structures and synthetic routes of 3,6-DMAC-AD-Py and 3,6-SFAC-AD-Py.

**General procedure for the synthesis of 3,6-DMAC-AD-Py and 3,6-SFAC-AD-Py**

To a mixture of 3,6-DF-AD-Py^3b^ (348 mg, 1.13 mmol), acridine (DMAC) or spiro-fluorene-acridine (SFAC) (2.49 mmol), Cs_2_CO_3_ (1.84 g, 5.65 mmol), DMF (18 mL) were added and heated to reflux for 24 h in N_2_ atmosphere. After cooling to room temperature, the solvent was distilled under vaccum, then extracted with dichloromethane (30 mL) and washed with saturated salt solution. The organic layer was evaporated under vacuum and the crude product was purified by column chromatography using dichloromethane as the eluent to obtain the light-yellow powder. The product was further purified by repeated recrystallization in dichloromethane/hexane to acquire the green-yellow solid.

**3,6-DMAC-AD-Py:** 427 mg, yield: 55%. ^1^H NMR (400 MHz, Chloroform-*d*) δ 8.85 (d, *J* = 8.5 Hz, 2H), 8.71 (d, *J* = 4.8 Hz, 1H), 7.97-7.93 (m, 1H),7.51-7.42 (m, 6H), 6.98-6.92 (m, 2H),7.04-6.87 (m, 8H), 6.62 (s, 2H), 630-6.27 (m, 4H), 1.63 (s, 12H). ^13^C NMR (101 MHz, CDCl_3_) δ 177.01, 152.24, 151.57, 146.62, 144.54, 140.85, 140.33, 131.19, 130.97, 126.61, 125.50, 125.27, 124.89, 124.23, 121.47, 121.19, 117.62, 114.97, 36.22, 31.23. HRMS (ESI, *m/z*): 687.3100 (calc. 686.3046). Elemental anal. Found: C, 83.97; H, 5.60; N, 8.14. Calcd for C_48_H_38_N_4_O: C, 83.94; H, 5.58; N, 8.16.

**3,6-SFAC-AD-Py:** 420 mg, yield: 40%. ^1^H NMR (400 MHz, Chloroform-*d*) δ 9.00 (d, *J* = 8.4 Hz, 2H), 8.86-8.85 (m, 1H), 8.13-8.09 (m, 1H), 7.80 (d, *J* = 7.6 Hz, 4H), 7.69-7.57 (m, 2H), 7.50-7.48 (m, 2H), 7.37 (t, *J* = 7.4 Hz, 4H), 7.30 (d, *J* = 7.5 Hz, 4H), 7.20 (d, *J* = 7.4 Hz, 4H), 6.97-6.88 (m, 4H), 6.83 (d, *J* = 1.7 Hz, 2H), 6.59 (t, *J* = 7.5 Hz, 4H), 6.45-6.34 (m, 8H). ^13^C NMR (101 MHz, CDCl_3_) δ 177.30, 156.34, 152.48, 151.63, 146.29, 144.67, 141.12, 140.68, 139.36, 131.34, 128.49, 128.03, 127.82, 127.54, 125.74, 125.42, 125.23, 125.20, 125.07, 121.91, 121.18, 120.12, 119.13, 114.72, 29.85. HRMS (ESI, *m/z*): 930.3364 (calc. 930.3359). Anal. Found: C, 87.74; H, 4.60; N, 6.00. Anal. Calcd for C_68_H_42_N_4_O: C, 87.72; H, 4.55; N, 6.02.

**2. Supplemental Tables and Figures**

**Table S1.** EL performance of 3,6-SFAC-AD-Py, 3,6-DMAC-AD-Py and representative TADF emitters with acridine derivatives reported in literatures.

| Compounds | *k*_r_  [10^7^ s^-1^] | *k*_RISC_  [10^6^ s^-1^] | EQE ^a^  [%] | Efficiency roll off [%] | λ_EL_  [nm] | CIE  (x, y) |  |
| --- | --- | --- | --- | --- | --- | --- | --- |
|  | 1.50 | 1.8 | 34.7/27.6 | 20 | 486 | 0.19, 0.37 | work |
|  | 0.98 | 1.0 | 32.4/23.4 | 28 | 496 | 0.22, 0.43 | work |
|  | 0.85 | 0.56 | 28.3/23.0 | 19 | 495 |  | [5] |
|  | 0.95 | 0.63 | 38.7/20.3 | 48 | 484 |  | [5] |
|  | 0.29 |  | 23.4/4.6 | 80 | 597 |  | [6] |
|  | 0.49 |  | 29.2/2.2 | 92 | 584 |  | [6] |
|  | 1.37 | 0.99 | 25.7/18.9 | 26 | 460 | 0.14, 0.15 | [7] |
|  | 0.22 | 6.42 | 28.4/26.8 | 6 |  |  | [8] |
|  | 1.0 | 0.21 | 28.2/17.6 | 38 | 456 | 0.142, 0.090 | [9] |
|  | 1.5 | 1.23 | 21.1/2.61 | 88 | 453 | 0.154, 0.046 | [10] |
|  | 0.8 | 1.7 | 21.6/10.8 | 50 | 436 | 0.146, 0.101 | [10] |
|  | 1.73 | 0.47 | 28.2 |  | 452 | 0.147, 0.092 | [11] |
|  | 1.84 | 0.56 | 31.2 |  | 448 | 0.149, 0.061 | [11] |
|  | 4.06 | 0.99 | 27.4/18.4 | 49 | 469 | 0.137, 0.147 | [12] |
|  | 4.68 | 0.67 | 31.5/17.6 | 44 | 466 | 0.132, 0.117 | [12] |
|  | 0.1 | 360 | 16.2/11.4 | 30 | 437 | 0.166, 0.066 | [13] |
|  | 0.2 | 410 | 25.4/20.0 | 21 | 444 | 0.151, 0.058 | [13] |
|  | 1.2 | 0.39 | 19.7/13.4 | 32 |  | 0.21, 0.50 | [14] |
|  | 4.6 | 0.63 | 36.7/30.5 | 20 |  | 0.18, 0.43 | [14] |
|  | 1.42 | 0.41 | 28.0/22.1 | 21 |  | 0.19, 0.42 | [4] |
|  | 1.24 | 0.78 | 37.3/31.7 | 15 | >500 |  | [15] |
|  | 1.74 | 0.88 | 32.6/21.1 | 35 | >500 |  | [16] |
|  | 1.57 | 0.97 | 35.8/24.7 | 31 | >500 |  | [16] |
|  | 1.24 | 0.11 | 32.0/20.1 | 37 | >500 |  | [16] |
|  | 1.11 | 0.11 | 27.2/18.2 | 33 | >500 |  | [17] |
|  | 3.28 | 0.74 | 25.6/15.0 | 36 | 496 | 0.200, 0.454 | [18] |
|  | 2.6 | 0.53 | 34.5/22.6 | 35 |  | 0.176, 0.374 | [19] |
|  | 2.6 | 0.6 | 30.5/20.0 | 34 |  | 0.182, 0.404 | [19] |
|  | 2.9 | 0.26 | 30.2/19.0 | 37 | 481 | 0.16, 0.30 | [20] |
|  | 2.2 | 0.19 | 27.2/17.7 | 35 | 493 | 0.17, 0.34 | [20] |
|  | 3.98 | 0.65 | 26.5/19.8 | 25 | 525 | 0.34, 0.54 | [21] |
|  | 3.91 | 0.67 | 29.6/19.5 | 34 | 510 | 0.28, 0.48 | [21] |
|  | 1.16 | 0.14 | 18.7 |  |  | 0.17,0.41 | [22] |
|  | 1.1 | 0.18 | 19.5/16.0 | 18 | 470 | 0.16, 0.20 | [23] |
|  | 0.5 | 0.5 | 26.9/19.4 | 28 | 484 | 0.14,0.14 | [24] |
|  | <1.0 | 1.2 | 22.4/17.3 | 23 |  | 0.16, 0.25 | [25] |
|  | 1.40 | 1.15 | 25/23 | 8 | 536 | 0.34, 0.59 | [26] |
|  | 1.70 | 0.85 | 27/26 | 4 | 510 | 0.25, 0.54 | [26] |
|  | 1.66 | 1.37 | 43.4/31.3 | 27.8 | 470 | 0.14, 0.18 | [27] |
|  | 0.4 | 2.5 | 17.34 |  | 537 | 0.37,0.55 | [28] |
|  | 0.6 | 2.22 | 8.53/5.12 | 40 | 612 | 0.582, 0.417 | [29] |

^a^ Maximum external quantum efficiency/external quantum efficiency a t 1000 cd m^-2^

**Table S2.** Calculated SOC matrix element values for emitters

| Compounds | <S_1_\|Ĥ_SOC_\|T_1_> [cm^-1^] | <S_1_\|Ĥ_SOC_\|T_2_> [cm^-1^] | <S_1_\|Ĥ_SOC_\|T_3_> [cm^-1^] | <S_2_\|Ĥ_SOC_\|T_1_> [cm^-1^] | <S_2_\|Ĥ_SOC_\|T_2_> [cm^-1^] | <S_2_\|Ĥ_SOC_\|T_3_> [cm^-1^] |
| --- | --- | --- | --- | --- | --- | --- |
| 3,6-DMAC-AD-Py | 0 | 0.170 | 0.933 | 0.085 | 0 | 0.283 |
| 3,6-SFAC-AD-Py | 0.05 | 0.184 | 1.111 | 0.178 | 0.03 | 0.450 |

**Figure S1.** Cyclic voltammograms of 3,6-DMAC-AD-Py and 3,6-SFAC-AD-Py.


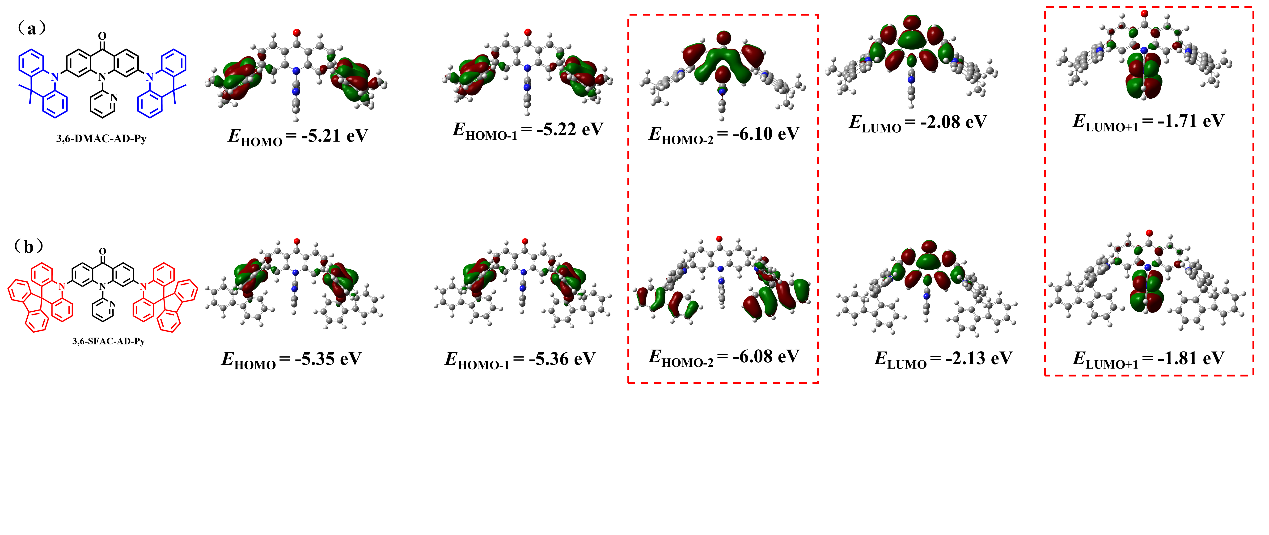


**Figure S2.** Chemical structures and FMOs distributions and levels for 3,6-DMAC-AD-Py (a) and 3,6-SFAC-AD-Py (b), respectively.


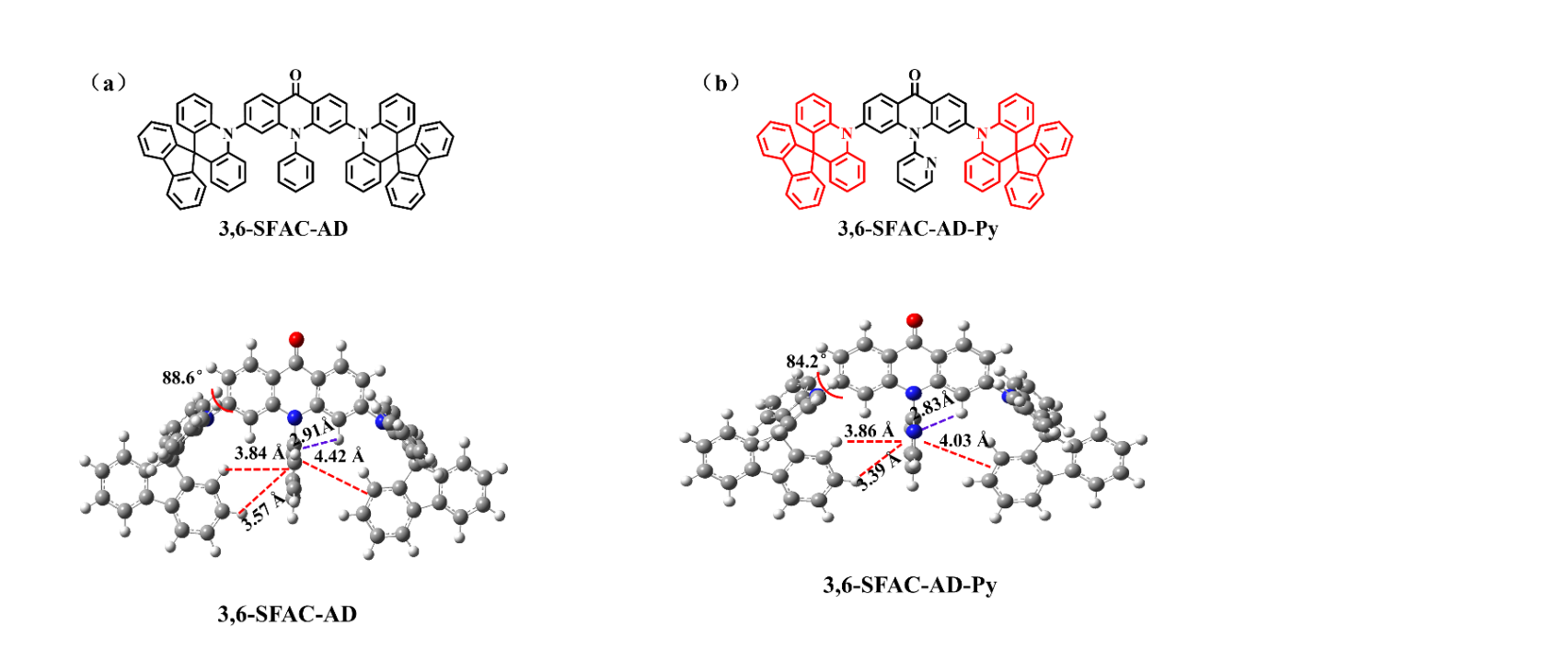


**Figure S3.** The chemical structures (up) and the optimized S_0_ geometries (down) of reference compound 3,6-SFAC-AD (a) and the target emitter 3,6-SAFC-AD-Py (b).

**Figure S4.** Chemical structure of the fragment compound AD-Py, and its low temperature fluorescence (LT-FL) and phosphorescent (PH) spectra in frozen 2-Me-THF solution at 77 K.

**Figure S5.** Transient PL decay curves of the doped films of these two TADF emitters in PPF host (7 wt%) at room temperature.

**Figure S6.** Delayed fluorescence spectra of these two TADF emitters in doped films (7 wt% in PPF) at room temperature.


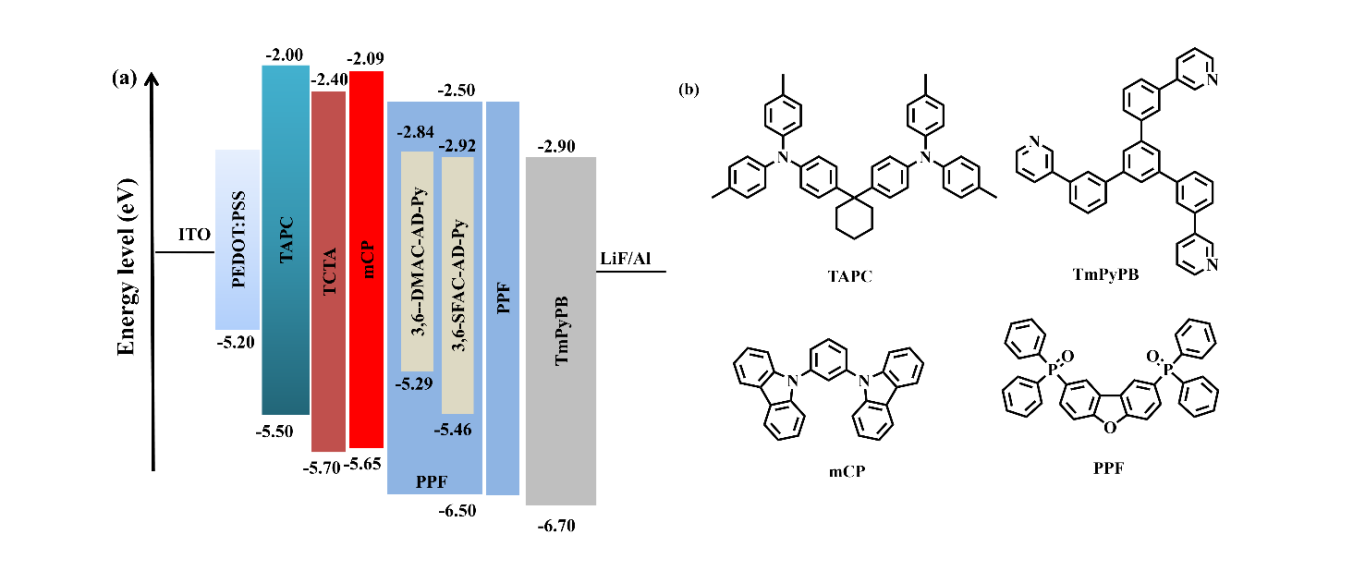

**Figure S7.** (a) Device architecture and energy diagram for the vacuum deposited OLEDs, (b) chemical structures of the relevant materials used for device fabrication, and (c) the *PE-B-CE* curves of device B1 and B2.

**References**

1. F. Neese, *WIREs Comput. Mol. Sci.* **2017,** 8, e1327.
2. T. Lu, F. Chen, *J. Comput. Chem.* **2012,** 33, 580-592.
3. (a) Y. Mei, D. Liu, J. Li, H. Li, W. Wei, J. Mater. Chem. C **2021,** 9, 5885-5892. (b) Y. Mei, Y. Lan, D. Li, J. Wang, L. Xie, X. Peng, J. Li, D. Liu, S.-J. Su, *Chem. Eng. J.* **2024,** 480, 148351.
4. L. Gan, Z. Xu, Z. Wang, B. Li, W. Li, X. Cai, K. Liu, Q. Liang, S.J. Su, *Adv. Funct. Mater.* **2019,** 29, 1808088.
5. G. Li, J. Pu, Z. Yang, H. Deng, Y. Liu, Z. Mao, J. Zhao, S.-J. Su, Z. Chi, Aggregate **2023,** e382.
6. Z. Wang, D. Li, W. Li, J. Zhang, M. Luo, S. Du, X. Zhang, S. Xu, Z. Ge, , *Adv. Opt. Mater*. **2021,** 11, 2300017.
7. D.H. Ahn, S.W. Kim, H. Lee, I.J. Ko, D. Karthik, J.Y. Lee, J.H. Kwon, *Nat. Photon*. **2019,** 13, 540-546.
8. Y.H. Lee, Y.-S. Shin, T. Lee, J. Jung, J.-H. Lee, M.H. Lee, *Chem. Eng. J.* **2021,** 423, 130224.
9. H. Lim, H.J. Cheon, S.J. Woo, S.K. Kwon, Y.H. Kim, J.J. Kim, *Adv. Mater.* **2020,** 32, e2004083.
10. H.J. Tan, G.X. Yang, Y.L. Deng, C. Cao, J.H. Tan, Z.L. Zhu, W.C. Chen, Y. Xiong, J.X. Jian, C.S. Lee, Q.X. Tong, *Adv. Mater.* **2022**, e2200537.
11. Y. Lee, J.-I. Hong, *Adv. Opt. Mater.* **2021,** 9, 2100406.
12. Y.H. Lee, J. Ji, T.Q. Tran, T. Lee, J. Jung, Y. Lee, S. Yoo, M.H. Lee, *Mater. Chem. Front.* **2023,** 7, 5413-5421.
13. G. Xia, C. Qu, Y. Zhu, J. Ye, K. Ye, Z. Zhang, Y. Wang, *Angew. Chem. Int. Ed.* **2021,** 60, 9598-9603.
14. T.A. Lin, T. Chatterjee, W.L. Tsai, W.K. Lee, M.J. Wu, M. Jiao, K.C. Pan, C.L. Yi, C.L. Chung, K.T. Wong, C.C. Wu, *Adv. Mater.* **2016,** 28, 6976-83.
15. R. Pei, Y. Xu, J. Miao, H. Peng, Z. Chen, C. Zhou, H. Liu, C. Yang, *Angew. Chem. Int. Ed.* **2023,** 62, e202217080.
16. H. Peng, Y. Xu, C. Zhou, R. Pei, J. Miao, H. Liu, C. Yang, *Adv. Funct. Mater.* **2023,** 33, 2211696.
17. H. Liu, Z. Liu, G. Li, H. Huang, C. Zhou, Z. Wang, C. Yang, *Angew. Chem. Int. Ed.* **2021,** 60, 12376-12380.
18. H. Peng, J. Lou, G. Li, C. Zhou, Z. Wang, H. Liu, *J. Mater. Chem. C* **2022,** 10, 5813-5820.
19. W. Li, M. Li, W. Li, Z. Xu, L. Gan, K. Liu, N. Zheng, C. Ning, D. Chen, Y.-C. Wu, S.-J. Su, *ACS Appl. Mater. Interfaces* **2021,** 13, 5302-5311.
20. W. Li, B. Li, X. Cai, L. Gan, Z. Xu, W. Li, K. Liu, D. Chen, S.J. Su, *Angew. Chem. Int. Ed.* **2019,** 58, 11301-11305.
21. Z. Xie, C. Cao, Y. Zou, X. Cao, C. Zhou, J. He, C.S. Lee, C. Yang, *Adv. Funct. Mater.* **2022,** 32, 2112881.
22. R. Yang, Q. Guan, Z. Liu, W. Song, L. Hong, T. Lei, Q. Wei, R. Peng, X. Fan, Z. Ge, *Chem. – An Asian J.* **2018,** 13, 1187-1191.
23. Q. Zhang, B. Li, S. Huang, H. Nomura, H. Tanaka, C. Adachi, *Nat. Photon.* **2014,** 8, 326-332.
24. J. Huo, S. Xiao, Y. Wu, M. Li, H. Tong, H. Shi, D. Ma, B.Z. Tang, *Chem*. *Eng. J.* **2023,** 452, 138957.
25. P. Stachelek, J.S. Ward, P.L. Dos Santos, A. Danos, M. Colella, N. Haase, S.J. Raynes, A.S. Batsanov, M.R. Bryce, A.P. Monkman, *ACS Appl. Mater. Interfaces* **2019,**11, 27125-27133.
26. J. Chen, J. Zeng, X. Zhu, J. Guo, Z. Zhao, B.Z. Tang, *CCS Chem.* **2021,** 3, 230-240.
27. Y. Fu, H. Liu, B.Z. Tang, Z. Zhao, *Nat. Commun*. **2023**, 14, 2019.
28. M. Ma, J. Li, D. Liu, D. Li, R. Dong, Y. Mei, *Dyes and Pigments* **2021**, 194, 109649.
29. S. Kothavale, K.H. Lee, J.Y. Lee, *ACS Appl. Mater. Interfaces* **2019**, 11, 17583-17591.
